# Genetic variant of *COL11A2* gene is functionally associated with developmental dysplasia of the hip in Chinese Han population

**DOI:** 10.18632/aging.103040

**Published:** 2020-05-12

**Authors:** Renjie Xu, Xin Jiang, Junlan Lu, Kexin Wang, Ye Sun, Yuxin Zhang

**Affiliations:** 1 Department of Rehabilitation Medicine, Kunshan Rehabilitation Hospital, Suzhou 210000, China; 2 Department of Rehabilitation Medicine, Shanghai Ninth People's Hospital Affiliated to Shanghai Jiao Tong University School of Medicine, Huangpu 200011, China; 3 School of Kinesiology, Shanghai University of Sport, Yangpu District, Shanghai 200438, China; 4 Clinical and Translational Research Center for 3D Printing Technology, Shanghai Key Laboratory of Orthopedic Implants, Department of Orthopedic Surgery, Shanghai Ninth People's Hospital, Shanghai Jiao Tong University School of Medicine, Shanghai 200011, China

**Keywords:** developmental dysplasia of the hip, COL11A2, single nucleotide polymorphism, gene expression, association study

## Abstract

Objectives: Developmental dysplasia of the hip (DDH) is a common skeletal disorder. This study was conducted to demonstrate the association between DDH and a polymorphism rs9277935 of *COL11A2* gene.

Results: A significant difference in genotype distribution in a recessive model (TT+GT vs. GG) between two groups (P=0.017) was demonstrated. Analysis in female patients showed significantly greater frequency of minor allele G(0.49 vs. 0.43, p=0.024) and significantly higher distribution of GG genotype (p=0.006). DDH patients were found to have significantly lower COL11A2 expression than controls. Moreover, DDH patients with rs9277935 genotype TT have a significantly increased expression of COL11A2 than those with genotype GG. COL11A2 demonstrated chondrogenic properties *in vitro*.

Conclusion: Polymorphism rs9277935 of gene *COL11A2* is a functional variant regulating the expression and the chondrogenic properties of COL11A2 in DDH in Chinese Han population.

Methods: A case-control candidate gene association study was conducted in 945 patients (350 radiologically confirmed DDH patients and 595 healthy controls). Difference of COL11A2 expression in hip joint tissue was compared between the patients and the controls. Allelic difference in Col11a2 expression by rs9277935 was assessed with luciferase activity. Chondrogenic effects of Col11a2 signaling on BMSCs were also determined *in vitro*.

## INTRODUCTION

Developmental dysplasia of the hip (DDH) (OMIM#142700) is a common congenital disease with both environmental and genetic components [1]. DDH patients present distinctively with hip joint laxity and skeletal dysplasia in the hip [2]. Subsequent dysfunction brings unacceptable life burden with skeletal development and physical growth [3]. DDH is a hereditary progression [4–6], though mechanical factors (e.g. breech delivery, high birth weight, primiparity and oligoamnios) are suggested [7, 8]. Several DDH susceptibility genes (e.g. GDF5, TBX4, ASPN, PAPPA2 and TGFB1) were discovered by association study in Chinese and Caucasian populations [9–13]. Gene mutations not only confer the sequence changes, but can also elicit certain structural change in the microenvironment [14, 15]. A variety of environmental factors have also been predicted to participate in DDH [16]. Yet the complex etiology of DDH remains to be clarified.

Various members (e.g. type II collagen, type XI collagen) in the collagen family are vital in skeletal development and connective tissue formation. Type XI collagen is composed of three subunits (α1, α2, α3) and expressed in bone, connective tissue and cartilage. It forms the major structure of articular cartilage and the core of collagen fibers together with type II collagen [17, 18]. *COL11A2* (collagen, type XI, alpha 2) gene (OMIM#120290) codes for the α2 subunit of type collagen XI. Mutations in *COL11A2* were reported to cause many inherited skeletal disorders like autosomal recessive otospondylomegaepiphyseal dysplasia (OSMED) (OMIM#215150) [19] and Marshall’s or Stickler syndrome (OMIM#184840) [20]. Several lines of evidence suggest that COL11A2 plays an important role in skeletal development. Nevertheless, there is no study of *COL11A2* concerning developmental dysplasia of the hip. In the present study, we have explored the association between *COL11A2* gene and DDH in the Han Chinese population.

## RESULTS

### Genetic association of locus rs9277935 in COL11A2 gene with DDH

Genotype distribution in both groups were conformed to Hardy-Weinberg equilibrium (p>0.01). Genotype and allele frequency distribution of Chinese Han population DDH group and healthy controls are shown in Table 1. The statistical analysis results indicate there is a trend of significant difference in allele frequency for the two groups (p=0.099). A significant difference in genotype distribution in a recessive model (TT+GT vs. GG) between two groups (P=0.017) was demonstrated. Due to the high prevalence of female patients, the genotype in the female from both groups was analyzed. (Table 2) There was significantly greater frequency of minor allele G in the female DDH patients (0.49 vs. 0.43, p=0.024). The recessive model also revealed significantly higher distribution of GG genotype in female DDH patients (p=0.006).

**Table 1:** Association between rs9277935 and DDH in different genetic models.

	**Genotype**			**Allele frequency**			**P value**		
	**T vs G**	**GG vs others**	TT vs others	**GG**	**GT**	**TT**	**No.**	**G**	**T**
**Case**	76	183	91	350	0.48	0.52			
**Control**	127	269	199	595	0.44	0.56	0.099	0.017	0.89

**Table 2:** Association between rs9277935 and DDH in the female population.

	**Genotype**			**Allele frequency**			**P value**		
	**GG**	**GT**	**TT**	**No.**	**G**	**T**	**T vs G**	**GG vs others**	**TT vs others**
**Female case**	71	163	78	312	0.49	0.51			
**Female control**	91	202	153	446	0.43	0.57	0.024	0.006	0.43

### Dysregulated joint tissue expression of the COL11A2 in DDH and the controls

**Figure 1:**
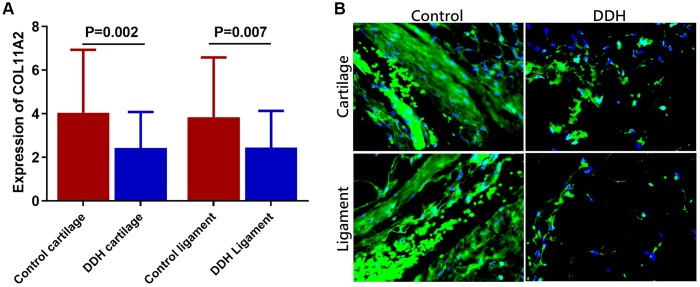
**Tissue expression of COL11A2 in patients and controls.** (**A**) DDH patients were found to have significantly lower expression of the COL11A2 in the articular cartilage and ligament as compared with the controls (2.43 ± 1.65 vs 4.05 ± 1.89, p = 0.002 for articular cartilage; 2.46 ±1.68 vs. 3.85 ± 2.73, p = 0.007 for joint ligament). (**B**) Immunofluorescent assay of COL11A2 (green) expression and nucleus (blue) in the cartilage and ligament tissues in different groups of patients observed under confocal microscopy.

Expression level of the COL11A2 was summarized in the patients and in the controls, respectively (Figure 1A). DDH patients had significantly lower expression of the COL11A2 in both joint cartilage and ligament compared with the control. (2.43 ± 1.65 vs 4.05 ± 1.89, p = 0.002 for articular cartilage; 2.46 ±1.68 vs. 3.85 ± 2.73, p = 0.007 for joint ligament). Moreover, immunofluorescent assay also demonstrated significantly lower COL11A2 expression in the tissue sections of cartilage and ligament tissues in DDH patients observed under confocal microscopy.

### Relationship between the genotype of rs9277935 and the COL11A2 expression

**Figure 2:**
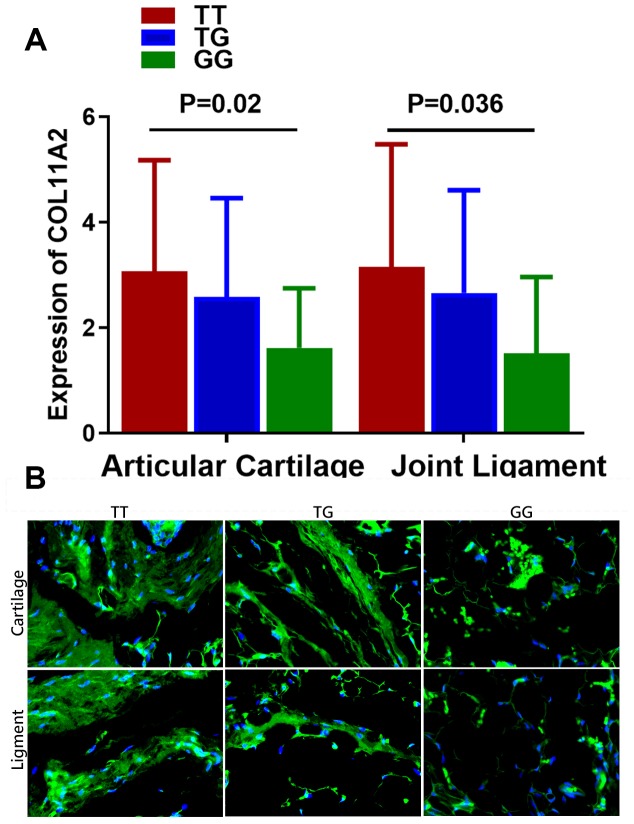
**Relationship between the genotype of rs9277935 and COL11A2 expression in DDH joint tissues.** (**A**) DDH Patients with genotype TT have a significantly increased expression of COL11A2 than those with GG (*p* = 0.02 for articular cartilage; *p* = 0.036 for joint ligament). (**B**) Immunofluorescent assay of COL11A2 (green) expression and nucleus (blue) in the cartilage and ligament tissues in DDH patients with different genotypes observed under confocal microscopy.

Results of the comparison of the COL11A2 expression among patients with different genotypes are shown in Figure 2A. The mean value of COL11A2 expression in articular cartilage and joint ligament were respectively 3.07 ± 2.11 and 3.15 ± 2.33 for genotype TT, 2.59 ± 1.87 and 2.66 ± 1.95 for genotype TG, and 1.62 ± 1.13 and 1.52 ± 1.45 for genotype GG. Patients with genotype TT were found to have a remarkably higher COL11A2 expression than those with genotype GG in both articular cartilage and joint ligament. (*p* = 0.02 for articular cartilage; *p* = 0.036 for joint ligament) Different COL11A2 expression was also demonstrated in tissue sections of cartilage and ligament tissues in DDH patients with different genotypes for rs9277935 with immunofluorescent assay, showing significantly greater COL11A2 expression in patients with TT genotype compared to those with GG genotype. (Figure 2B).

### Allelic difference in Col11a2 expression by rs9277935 and the chondrogenic effects of Col11a2 on BMSCs *in vitro*

**Figure 3:**
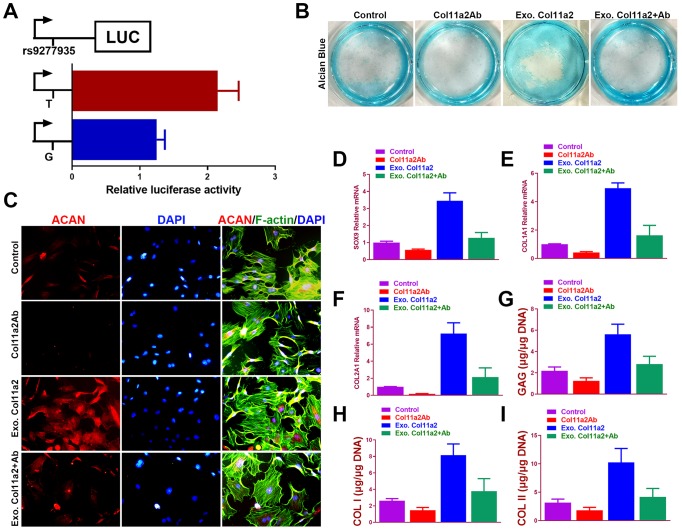
**Chondrogenic effects of Col11a2 *in vitro*.** (**A**) Luciferase activity to indicate the allelic difference in Col11a2 expression driven by rs9277935 (n=6 for each) in ATDC5 cells. (**B**) Alcian blue staining of BMSCs in different treatment groups at 2 weeks to indicate GAG production in the culture plate. (**C**) Immunofluorescent assay of Aggrecan (red) expression, cytoskeleton (green) nucleus (blue) in different treatment groups observed under confocal microscopy. Scale bar=200μm. (**D**–**F**) Expression level of chondrogenic markers for BMSCs in different treatment groups (n=6 for each) in (**B**) and (**G**–**I**) quantification of GAG production, COL I and II DNA in generated cartilaginous tissues (n=6 for each). *P < 0.05 between control group and other groups. Data are presented as averages ± SD. One-way analysis of variance (ANOVA) with post-hoc Tukey’s B test was applied. Ab, antibody; Exo, exogenous; ACAN, aggrecan; GAG, glycosaminoglycan.

To verify the allelic difference in Col11a2 expression by locus rs9277935 we tested the luciferase activity driven by different alleles. (Figure 3A) In comparison, it was apparent that T allele drove greater luciferase expression than G, with a 40% difference seen in ATDC5 (Figure 3A), indicating rs9277935 as a causative locus driving the luciferase expression change. These data clearly demonstrate that the rs9277935 variant associated with DDH were functional and mediated dysregulated Col11a2 expression, which partly explained the decreased Col11a2 expression in DDH. To test the involvement of dysregulated Col11a2 expression in chondrogenesis, potential chondrogenic effects of Col11a2 on chondrogenesis of bone marrow stem cells (BMSCs) were further analyzed *in vitro*. (Figure 3B to I) Addition of Col11a2 blocking antibody lowered production of glycosaminoglycans (GAGs) while application of exogenous recombinant Col11a2 (50ng/ml) for 2 weeks in culture induced greater synthesized glycosaminoglycans (GAGs) with strong Alcian blue staining, indicating differentiation of BMSCs into chondrocyte-like cells. The chondrogenic effects of exogenous Col11a2 was further evidenced by its neutralization in the group with Col11a2 blocking antibody. Chondrocytes generated in the several groups were next analyzed with immunostaining and RT-PCR (Figure 3C–3I) for expression of genes expressed by chondrocytes (ACAN, SOX9, COL1A1, COL2A1). Compared to the control, exogenous Col11a2 treatment led to significant chondrogenic effects evidenced by chondrogenic gene expression (Figure 3C–3F) and GAG deposition with higher DNA quantification of collagen I and II (Figure 3G–3I). The chondrogenic effects of Col11a2 on BMSCs were blocked with supplemented Col11a2 blocking antibody in the other two groups. These results indicated that Col11a2 as an important mediator in chondrogenesis and cartilage formation.

## DISCUSSION

In the present study, we first explored the association between *COL11A2* gene and DDH. The minor allele frequency of rs9277935 in the control group in our study (0.44) is close to that reported in HapMap for Chinese Han population (0.47). A trend of difference of allele frequency between DDH and control group was demonstrated. Whereas a significant difference of) genotype distribution in a recessive model (TT+GT vs. GG between two groups was identified. Further analysis in female population showed significant difference in both genotypes and allele frequency distributions, suggesting association between rs9277935 in gene COL11A2 and DDH occurrence in female patients. DDH patients were found to have a significantly lower expression of COL11A2 in both the cartilage and ligament than the control groups. Besides, patients with genotype TT of rs9277935 were found to have a remarkably increased expression of COL11A2 than those with GG. Allele G is indicative of lower expression of COL11A2 as well as a higher risk of the development of DDH. Taken together, rs9277935 could be a functional variant regulating the expression of COL11A2.

DDH is a complex polygenic disease, whereby multiple susceptibility genes have been reported. Jia et al reported that rs726252 in pregnancy - associated plasma protein - A2 gene, with significantly greater distribution of TT genotype in DDH patients, was associated with DDH [10]. The research conducted by Shi et al showed an obvious association between the D repeat polymorphism of ASPN and DDH, which indicated that ASPN is an important regulator in the etiology of DDH [11]. In addition, Wang et al discovered that a single nucleotide polymorphism in Tbx4 was also associated with DDH [12]. Furthermore, Jin et al found that GDF5 is important in the etiology of congenital dysplasia of the hip [13]. A recent study showed that TGFB1 played a genetic role in the risk of DDH [9]. All of these indicated the complex genetic components in DDH occurrence. DDH patients usually present distinctively with laxity of joint capsule, skeletal dysplasia of the femoral head and acetabular dysplasia. However, the patients retrieved in the present study were mostly the elder DDH patients with secondary arthritis. In this case, the exact primary cause of DDH was difficult to determine in these patients. Further studies of DDH in infants or adolescents could help to address this issue.

*COL11A2* is an indispensable gene involved in collagen formation and extracellular matrix building, which are fundamental in development of cartilage and articular joint, shaping of joint morphology and biomechanical balance maintaining [21–24], indicating a possible role of *COL11A2* in DDH development. This gene codes for type collagen XI, which is expressed in cartilage and bone. Collagen type XI constitutes the major structure of articular cartilage with collagen type II. It is wildly expressed in cartilage as a target gene downstream of Sox9 [25]. While Sox9 gene haplo-insufficiency can lead to genetic skeletal disorders like campomelic dysplasia (CMD) (OMIM#114290) characterized by bowing long bones [26, 27]. *COL11A2* mutations are also associated with a lot of inherited skeletal diseases, whereby Otospondylomegaepiphyseal dysplasia (OSMED) is autosomal recessive disease, featuring auditory dysfunction and short asymmetrical limbs [28]. Mutations of *COL11A2* can also cause an skeletal dysplasia called fibrochondrogenesis (OMIM#614524) [29]. Moreover, Pihlajamaa et al reported a single nucleotide mutation in *COL11A2* gene that converted glycine to glutamate (G955E) and caused Weissenbacher-Zweymuller syndrome (OMIM#277610) [30]. One C to T transition in exon 56, which led to premature translation termination codon in *COL11A2* (R845X), was also reported in individuals with Weissenbacher-Zweymuller syndrome [31]. In addition, abnormal *COL11A2* gene structure was also associated with Marshall’s or Stickler syndrome marked with skeletal dysplasia and joint pain [32].

Close relationship between *COL11A2* gene and the aforementioned skeletal disorders is suggestive of its significant role in bone and joint development. This present study was the first report concerning *COL11A2* in developmental dysplasia of the hip to date. The locus rs9277935 was formerly reported to associate with autoimmune disease (e.g. Wegener's granulomatosis) [33]. rs9277935 locates in the promoter region of gene COL11A2 and modifies the expression of COL11A2 as demonstrated by the allelic difference of COL11A2 expression driven by rs9277935 in the present study. It is possible that rs9277935 affected the binding of transcript factors or other motifs to the promoter of COL11A2 gene to regulate its final expression. In clinical practice, DDH patients usually present with laxity of joint capsule and chondrogenic dysplasia of the femoral head. Arthritic changes occur in most of DDH patients, afflicting mainly the chondrocytes of the femoral head. COL11A2 was an indispensable gene in chondrogenesis and rs9277935 modulated COL11A2 in the present study in DDH patients. Therefore, we further validated the chondrogenic role of COL11A2 in studies *in vitro*, suggesting that altered chondrogenesis with changed COL11A2 signaling by the variant rs9277935 could be the causative role in DDH. The association between rs9277935 and DDH was mediated by dysregulated chondrogenesis and joint cartilage formation caused by the allelic difference of COL11A2 gene expression. Experiments *in vivo* to further explore the function of *COL11A2* in DDH occurrence is necessary.

## CONCLUSIONS

In conclusion, we demonstrated the locus rs9277935 of the chondrogenic gene *COL11A2* was functionally associated with DDH with dysregulated COL11A2 expression in hip joint tissues in Chinese Han population. Genotype TT of rs9277935 is associated with higher expression of COL11A2 in DDH. Genetic variants of COL11A2 could be a potential biomarker for early diagnosis of DDH. Replication work in other ethnic populations is needed.

## MATERIALS AND METHODS

### Patients

We enrolled 350 radiologically confirmed DDH patients and 595 healthy controls to conduct a case-control candidate gene association study. DDH patients were consecutively recruited from the department of orthopedics, the first affiliated hospital of Soochow University. Controls were recruited from physical examination center in the first affiliated hospital of Soochow University. All the subjects were Han Chinese living in or around Suzhou. The study was approved by the ethical committee of the Soochow University, and informed consents were obtained from all patients and controls.

### Sample collection

After informed consent was obtained from the families, blood samples were collected for genomic DNA extraction using the commercial kit (QIAGEN) according to the standard protocol. Hip joint articular cartilage and ligament were obtained in 65 DDH patients during hip arthroplasty surgery. 20 age-matched trauma patients undergoing amputation surgery were recruited as control. Tissue samples were placed in separate sterile tubes and immediately stored in liquid nitrogen. Frozen samples were stored at -80 °C. Total RNA was extracted using Trizol reagent (QIAGEN) according to the manufacturer’s protocol. To avoid genomic DNA contamination in RNA, samples were treated with DNase (QIAGEN). Total RNA was then reverse-transcribed from 2ug of RNA using the PrimeScript RT Master Mix kit (TaKaRa).

### Genotyping of targeted locus

According to the manufacture’s protocol, the DNA of all the subjects was extracted either from the buccal swabs using the DNA IQ System (Promega, Madison, WI) or peripheral blood using the NucleoSpin Blood QuickPure Kit (Macherey-Nagel GmbH & Co. KG, Düren, German). All the samples were genotyped with Taqman assay. The sample was genotyped by uninformed laboratory personnel. Genotyping, data input and statistical results were examined by two authors independently. Five percent of samples were randomly selected to repeat, and 100% consistency was obtained.

### Tissue expression of the *COL11A2* gene in DDH patients and controls

The tissue expression of *COL11A2* was measured with real-time PCR using gene-specific primers as follows: forward 5’-GCCTCAGCCTAGCAGAT -3’, reverse 5’- ATCACTCCATGGGTGTCCAATA -3’ for the *COL11A2* gene, and forward 5’-GAGTC AACGGATTTGGTCGT-3’, reverse 5’-TTGATTTTGGAGGGATCTCG-3’ for the endogenous control gene Glyceraldehyde-3-phosphate dehydrogenase (*GAPDH*). For real-time PCR, 1 μL of cDNA was amplified for 40 cycles by SYBR.

Premix Ex TaqTM II (TaKaRa) in ABI 7900HT mentioned above. Melting curve analysis was done at the end of the reaction to assess the quality of the final PCR products. All samples were analyzed in triplicate using the 2^-ΔΔCt^ method.

### Immunofluorescent staining of histological sections

Frozen sections of hip joint tissue were fixed with 4% paraformaldehyde for 5 min. After washing with TBS-T, the sections were incubated with 10 mg/mL hyaluronidase (Sigma) at 37 °C for 30 min. After blocking with 10% goat normal serum, the sections were incubated with primary antibody for 2 hat room temperature. Paraffin-embedded sections were deparaffinized and incubated in 1mM EDTA (pH 8.0) at 80 °C for 15 mins to retrieve the antigen. Then sections were treated with 10 mg/mL hyaluronidase at 37 °C for 30 min. The primary antibodies anti-COL11A2 were used (diluted 1:200, Abcam). Immune complexes were detected using secondary antibody. DAPI was also used to detect the nucleus.

### Construction of COL11A2-pGL3-basic luciferase reporter plasmids

COL11A2-pGL3-basic luciferase reporter plasmids for rs9277935 were constructed as previously reported [2]. Briefly, ATDC5 cells were transfected using 500 ng of pGL3 plasmid DNA and 15 ng of pTK-RL Renilla plasmid in combination with Exgen 500 Transfection reagent (Fermentas, Sankt Leon-Rot, Germany) [34–36]. Twenty-four hours after transfection ATDC5 were lysed, using passive lysis buffer (Promega), and the protein extracted and stored at -20 °C. Lysate sample was mixed with luciferase activating reagent II (Promega) and a 1-s luciferase activity reading was measured using a luminometer (EG&G Berthold, Bad Wildbad, Germany). Stop and Glo (Promega) was added to each sample to measure renilla activity. An empty vector transfection was performed as a control. Each experiment contained six replicates, and was repeated three times, producing a total of 18 data points. A Student’s t-test was performed to assess any significant differences in luciferase activity between the different alleles.

### Isolation and treatment of BMSC *in vitro*

Mouse BMSCs were isolated from rabbit bone marrow aspirates [37]. Briefly, marrow aspirates (20 mL volume) were harvested and immediately transferred into plastic tubes. Isolated BMSCs were expanded in α-MEM containing 10% FBS, 4.5 mg/mL D-glucose,0.1 mM nonessential amino acids, 1 mM sodium pyruvate, 100 mM Hepes buffer, 100 Ul/mL penicillin, 100 μg/mL streptomycin, and 0.29 mg/mL L-glutamate. Medium was changed twice a week and BMSCs were used at P2 for the following experiments [34, 38]. In the exogenous COL11A2 group, COL11A2 (50 ng/ml) was added in the medium for 2 weeks. COL11A2 neutralizing peptide was added in some of the cultures according to the protocol. Medium was also changed twice a week.

Immunofluorescence staining of chondrogenic marker ACAN was conducted to compare the phenotypes of generated chondrocytes in different groups and observed under confocal microscopy (Leica, Japan). The expression of chondrogenesis marker (SOX9, Col1A1 and Col2A1) after 2-week incubation was analyzed by real-time polymerase chain reaction (RT-PCR) using an ABI 7300 RT-PCR system (Applied Biosystems, USA). Two-week-old tissue generated under different conditions was observed and stained with alcian blue for proteoglycan production. The stained images were taken using a light microscope (Leica Microsystems, Germany).

GAGs and types I and II collagen were quantitatively assayed (6 vs 6) and normalized to DNA content. GAG production, Collagen I and II expression were compared among different treatment groups.

### Statistics

Hardy-Weinberg equilibrium was calculated by chi-squared test in both control and case groups. The association between the DDH patients and the control subjects in the stages was tested by SAS software (version 9.2 - SAS Institute, Cary, NC, USA). Bilateral chi square tests were conducted to determine the significance of differences in allelic frequencies and P < 0.05 was considered to be statistically significant. The Student t test was used to compare the difference of COL11A2 expression between the patients and the controls. DDH patients were classified into three groups according to the genotypes of each SNP, and One-way ANOVA test was used to compare the COL11A2 expression among different genotypes.
